# Effects of breast stimulation for spontaneous onset of labor on salivary oxytocin levels in low-risk pregnant women: A feasibility study

**DOI:** 10.1371/journal.pone.0192757

**Published:** 2018-02-15

**Authors:** Kaori Takahata, Shigeko Horiuchi, Yuriko Tadokoro, Takuya Shuo, Erika Sawano, Kazuyuki Shinohara

**Affiliations:** 1 St. Luke’s International University, Tokyo, Japan; 2 Graduate School of Nursing Science, St. Luke’s International University, Tokyo, Japan; 3 St. Luke’s Maternity Care Home, Tokyo, Japan; 4 Hokuriku University, Ishikawa, Japan; 5 Department of Neurobiology and Behavior, Graduate School of Biomedical Sciences, Nagasaki University, Nagasaki, Japan; VU medisch centrum, NETHERLANDS

## Abstract

**Objectives:**

This preliminary study aimed to 1) determine changes in the salivary oxytocin (OT) level during breast stimulation for promoting the spontaneous onset of labor in low-risk term pregnancies, and 2) clarify the feasibility of the breast stimulation intervention protocol in terms of practicality and acceptability.

**Methods:**

We used a single arm trial design. Sixteen low-risk pregnant women between 38 and 40 weeks of gestation with cephalic presentation participated. They performed breast stimulation for 3 days with an attendant midwife in a single maternity hospital. Each breast was stimulated for 15 minutes for a total of 1 hour per day. Saliva was collected 10 minutes before the intervention and 15, 30, 60, 75, and 90 minutes after the intervention, yielding 18 samples per woman.

**Results:**

Among a total of 282 saliva samples from the 16 participants, OT level was measured in 142 samples (missing rate: 49.6%). The median OT level showed the highest values on day 3 of the breast stimulation, with a marked increase 30 min after the intervention. In the mixed models after multiple imputation for missing data, the OT level on the first day of intervention was significantly lower than that on the third day of intervention. Fatigue from breast stimulation decreased on subsequent days, and most of the women (75%) felt no discomfort with the protocol. Uterine hyperstimulation was not observed.

**Conclusion:**

Following a 3-day breast stimulation protocol for spontaneous onset of labor, the mean OT level showed the highest values on day 3. The breast stimulation intervention protocol showed good feasibility in terms of practicality and acceptability among the pregnant women. Additional large-scale studies are warranted to confirm the protocol’s effectiveness.

## Introduction

Oxytocin (OT) infusion is one of the most commonly used techniques for labor induction to avoid increased maternal and fetal risks beyond term [1]. However, labor induction is a relevant negative birth experience for women [2,3]. There is also speculation regarding the possible association between the use of synthetic OT and the development of autism, although no definite conclusion has been reached regarding the long-term adverse events of synthetic OT [4,5]. In Japan, about 50% of pregnant women perform exercise or breast stimulation to help induce spontaneous labor [6].

The effects of complementary and alternative medicine on labor induction are expected during membrane sweeping and breast stimulation [7,8]. In particular, breast stimulation has historically been used to induce and augment labor [9]. It is a natural method that requires no cost and tool, and it can be performed at any time and place depending on a pregnant woman’s own initiative. Breast stimulation for inducing labor has been studied by many researchers to date [10–16].

In the systematic review analysis of 6 trials consisting of 719 women by Kavanagh et al. involving a comparison of the effect of breast stimulation with no intervention, they found a significantly low number of women who were not in labor at 72 hours after the stimulation (62.7% vs 93.6%; relative risk, 0.67; 95% confidence interval, 0.60 to 0.74). The number needed to treat was only 4, indicating that effective intervention can still be achieved even with small numbers. The minimum breast stimulation time to induce spontaneous labor was reported to be 1 hour each day for 3 days [10].

Several mechanisms underlie the onset of labor, which appears to be associated with different plural factors. In particular, the underlying mechanism of breast simulation inducing the onset of labor is considered to be associated with the OT level.

In the 1980s, the most frequently investigated procedure for the contraction stress test (CST) was the induction of OT release by breast stimulation in pregnancy instead of the use of synthetic OT. CST is an examination method for determining the coping ability of the fetus with uterine contractions mainly for high-risk pregnancies. It is one of the antepartum fetal surveillance assessment techniques. Amico et al. reported that several minutes of CST by breast stimulation in the third trimester increased plasma OT level [17]. On the other hand, Ross et al. showed that CST by breast stimulation failed to significantly increase plasma OT level [18]. Thus far, the results obtained remain controversial, and little has been reported about OT levels following breast stimulation for 3 days to promote spontaneous onset of labor.

The objectives of this preliminary study were to 1) determine the changes in the salivary OT level during breast stimulation for promoting the spontaneous onset of labor in low-risk term pregnancies, and 2) clarify the feasibility of the breast stimulation intervention protocol used in terms of its practicality and acceptability.

## Materials and methods

### Study design

This trial used a quasi-experimental single-arm time series design.

### Participants and setting

**Fig 1:**
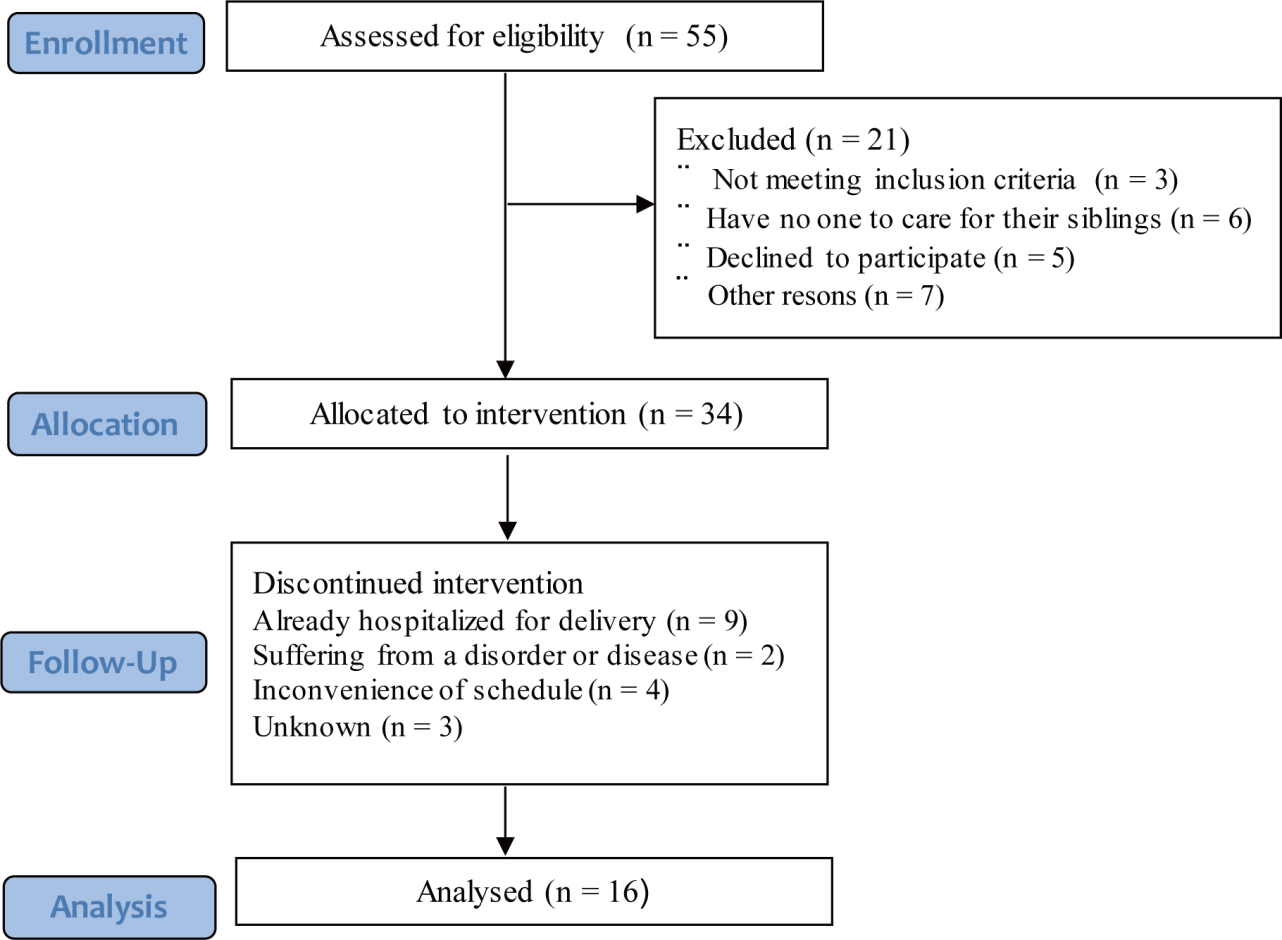
Participant flow diagram.

This research was a feasibility study. The sample size was based on previous research reporting on saliva OT level from 11 breastfeeding women [19]. Previous studies reporting on plasma OT level in pregnant women involved about 10 to 20 subjects [17,18,20–22]. The eligibility criteria were as follows: planned to give singleton birth by spontaneous cephalic delivery; between 38 and 40 weeks of gestation; Asian and can read and write Japanese; received permission from the obstetrician or midwife to participate. The exclusion criteria were as follows: taking any medications related to their gestation; have medical or pregnancy complications and mental illness; have a medical history of Assisted Reproductive Technology treatment; a pre-pregnancy BMI > 25; planned induced labor; experienced prolonged pregnancy; had previous caesarian section; breastfeeding a child. A flowchart demonstrating the recruitment of the study participants is shown in Fig 1.

When eligible pregnant women at 34 weeks of gestation visited the outpatient ward for a prenatal check-up, we verbally explained our research in a place without a nurse or an obstetrician. After obtaining consent, we decided with the participant the first intervention day after 38 weeks of gestation. Women also received a refusal form at that time with an explanation that they can withdraw any time without any disadvantage. In the data collection facility of this study, pelvic examination is performed for all pregnant women after the 37 weeks check-up. Membrane sweeping may be performed at the discretion of an obstetrician after 39 gestational weeks.

The experiments were held in the hospital for 3 consecutive days. There were some subjects who had perinatal check-ups by obstetricians before and after the experiment. Data were collected at a single maternity hospital in Kanagawa, Japan between June 2015 and August 2015.

The study protocol was approved by the Institutional Review Board of St. Luke’s International University, Tokyo, Japan (No. 15–011). This study was registered in the Clinical Trials Registry of University Hospital Medical Information Network in Japan (UMIN000017911).

### Procedures

The breast stimulation intervention time was 3 days. The same time of the day was used to control for diurnal effects. Saliva was collected within the period from 10:00 to 16:00 hrs. The participants were asked to refrain from sexual intercourse and drinking alcohol before the day of intervention. They were also instructed to finish their meal, brush their teeth, and not to smoke 1 hour before the intervention. They were also asked not to use a lipstick. The experiment was started 30 minutes after the intake of 100 mL of water.

**Fig 2:**
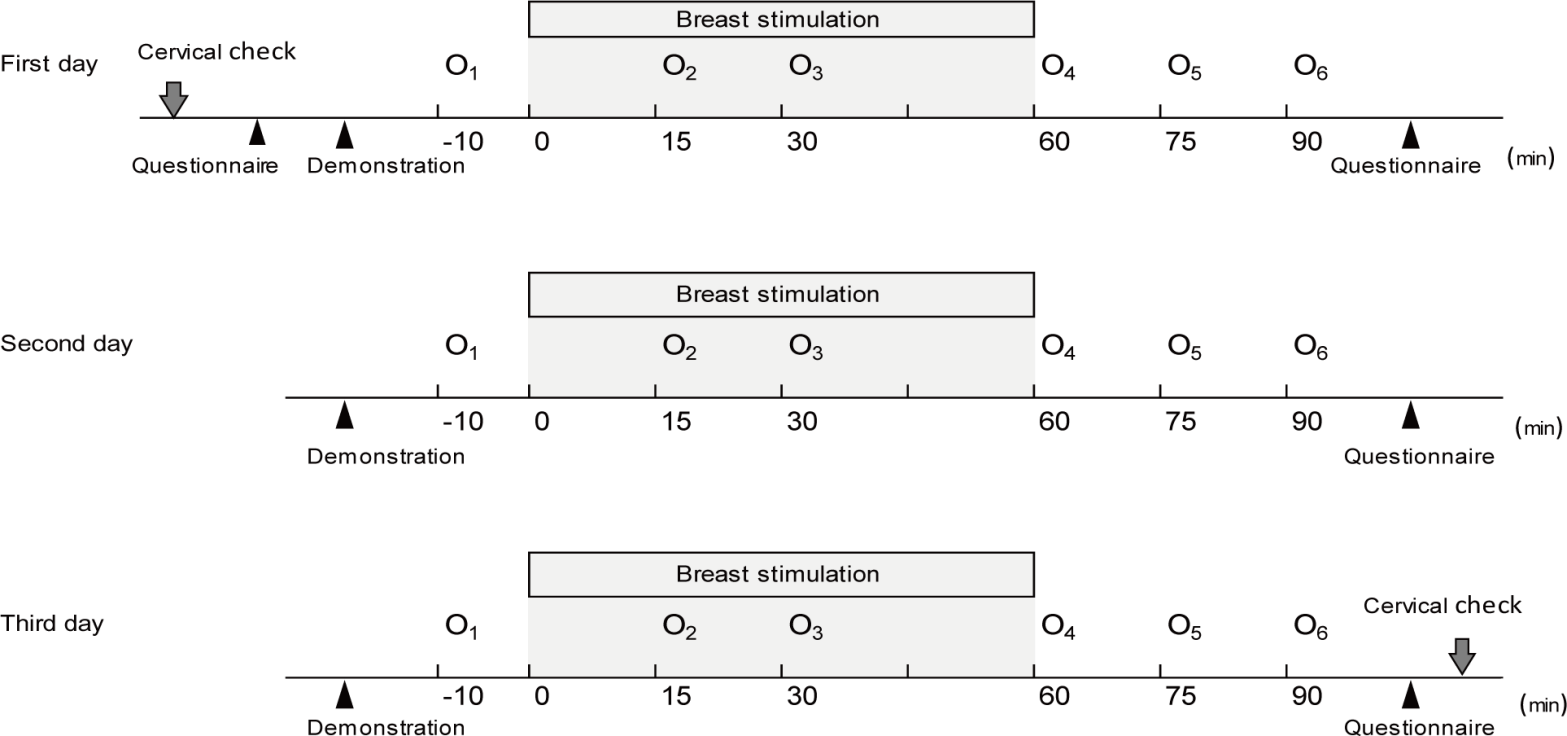
Intervention procedure for days 1–3.

At the start of the study, the cervix score was evaluated for cervical ripening (**Fig 2**; cervical check) using a modified Bishop Score. Cervical ripening can be a possible option for evaluating the intervention in relation to the endpoint. The evaluators were composed of 3 midwives who have 10 years of experience to standardize the assessment.

The participants performed breast stimulation for 3 days together with an attendant midwife in the hospital. To standardize the intervention technique, a midwife demonstrated how to perform breast stimulation for 10 minutes using a breast model prepared from wool and a pressure-measuring instrument (Perineometer, OWOMED, Gyeonggido, South Korea). The recommended stimulation pressure was between 20 and 50 mmHg, which is a gentle pressure and does not damage the nipple. To achieve a stimulation rhythm of 69 beats per minute, a lighting electric metronome (SQ50V, Seiko Watch Corporation, Tokyo, Japan) without a sound was used as guide. Each breast was stimulated for 15 minutes, beginning on the right, alternating between the breasts to prevent uterine hyperstimulation, for a total of 1 hour per day. Breast stimulation was performed by pinching the breast to a degree that did not hurt starting from the outer areola to the nipple using the thumb, forefinger, and middle finger. Direct self-stimulation of the breast was performed using either the left or right hand with Pure Horse Oil (Sonbahyu; Yakushido, Fukuoka, Japan). To address concerns or worries regarding the presence of others during breast stimulation, a nursing cover was used. We managed the time and notified the participants.

Cardiotocography (CTG) readings were obtained during the intervention to record uterine activity and check the fetal heart rate. Adequate uterine contraction was defined as having at least 3 contractions lasting for 40 seconds each occurring within 10 minutes. The frequency of uterine contraction can also be a possible option for evaluating the intervention in relation to the endpoint. The participants watched a prepared movie (i.e., train tours across Europe) to control external factors that may affect the experimental environment. After the intervention, the participants answered a questionnaire on the feasibility of the experimental intervention. Cervical ripening was reassessed at the end of the intervention.

After the experimental intervention, all the participants received a fee by bank transfer for participating in the study (\3,000 or about$30).

The intervention was performed by 3 midwives consisting of the first author and 2 research assistants. They had adequate CTG reading ability gained from their more than 5 years of clinical experience and over 100 cases of conducting labor management for this feasibility study. For the intervention standardization, the explanations for the participants and intervention details were unified. Moreover, the first author consistently participated in the experiments twice at the beginning to ensure that the research assistants can properly implement the intervention procedure. Research assistant A was responsible for 30.3% of the experiments, B for 3.0%, and the first author for the rest of the experiments. In addition, in 40% of the days when research assistant A was responsible for the experiments, the first author was in the hospital and confirmed the standardization of the experiments.

### Saliva collection procedure

Salivary assays are relatively easy to perform and are minimally invasive for pregnant women. A moderate correlation (r = 0.41–0.59) has been reported between salivary OT level and plasma OT level [23–25]. Moreover, a positive correlation (r = 0.89) has been shown between salivary OT and plasma OT in patients with no history of self-induced vomiting in anorexia nervosa [26].

Saliva samples were collected 10 minutes before and 15, 30, 60, 75, and 90 minutes after the breast stimulation intervention for a total of 18 samples per participant. One milliliter of saliva was collected by unstimulated passive drool for the measurement of OT level by enzyme-linked immunosorbent assay (ELISA; ENZO Life Sciences, NY, USA). The ELISA manual states that the intra-assay and inter-assay coefficients of variability are 12.6% - 13.3% and 11.9% - 20.9%, respectively.

To standardize the saliva collection procedure, the participants were asked to pool their saliva in their mouths for 3 minutes. Afterwards, the participants collected about 1.0 mL of their saliva for each sample in a 1.5 mL polypropylene tube (Eppendorf, NY, USA) using a saliva collection aid (Salimetrics, PA, USA). If this volume could not be collected after 3 minutes, the participants repeated the saliva collection procedure. The collected samples were immediately stored in a freezer (Cryo Porter CS-80C, Scinics Corp., Tokyo, Japan) at -80°C. OT level was assayed using the method of Carter et al. [27]. For other experimental processes, we added 500 KIU/μL aprotinin after thawing to prevent proteolytic degradation.

### Polymorphisms of oxytocin receptor

OT receptor single nucleotide polymorphism has been reported to reduce sensitivity to OT [28,29]. In particular, women with GG homozygous types at OT receptor gene rs53576 reportedly transitioned to late active labor. In the present study, buccal mucosa samples were obtained for the analysis of 3 OT receptor gene polymorphisms (i.e., rs53576, rs2254298, and rs1042778) by genotyping using TaqMan SNP assay (Applied Biosystems, Thermo Fisher, MA, USA).

### Feasibility in terms of practicality and acceptability

We evaluated the feasibility of the breast stimulation intervention protocol in terms of practicality and acceptability [30]. For the *practicality of the experimental method*, the proportion of analyzable samples and the occurrence of adverse events were considered. For the *acceptability of the experimental method*, the dropout rate after the intervention was considered. The visual analog scale (VAS), which is one of the most valid, reliable, and frequently used measurement tools for a self-report measure, was used in the present study to rate fatigue [31] and pain [32]. Women responded to the following comments and questions using the following scales [from 0 (no fatigue) to 100 (fatigue)] or [from 0 (no pain) to 100 (pain)]:“Please give your feedback regarding the breast massage; 1) Did your arms and fingers get fatigued? 2) Did you have any pain in the nipple?” and “Please give your feedback regarding the saliva collection; 1) Did you experience fatigue from the saliva collection?”. A 4-level Likert scale [1 (strongly agree) to 4 (strongly disagree)] was used when answering the following questions: 1) “Was it uncomfortable touching the nipple?”, 2) “Was the instruction for the stimulation method easy to understand?”, 3) “Do you want to do this procedure again in your next pregnancy?”, and 4) “Would you like to provide information about this technique to your friends?”. Considering the cultural background of East Asians tending to choose the midpoint [33,34], a 4-level Likert scale was used for the measurement of attitude to avoid ambiguity for these questions. On the other hand, for the rhythm and intensity of the stimulation, a 5-level Likert scale was used, which includes “moderately” as an intermediate choice. In addition, the participants responded to questions about the environment where the intervention was carried out.

### Data analysis

The means (*M*), standard deviations (*SD*), standard errors (*SE*), and medians of all variables were calculated. We employed a linear mixed model using the AR (1) covariance structure with the OT level as the dependent variable and the day and time of intervention as the fixed effects. Little’s MCAR test was performed. In addition to all the OT levels, age, gestational weeks at the intervention, obstetric history, marital status, living with partner, and education ≥ 12 years were included in the imputation procedure as factors influencing the OT level. The result was *p* = 0.980, and the null hypothesis was not rejected. The main cause of the missing data was insufficient saliva volume and not missing data due to the OT value, thus the missing data in this study reflected *missing completely at random* (MCAR). We used the multiple imputation procedure in SPSS Missing Values to impute the missing data. Five imputed datasets were created. The data had a non-normal distribution. Spearman’s correlation coefficient was used to test the correlation between salivary OT level and cervical score or characteristics. Statistical analyses were performed using IBM SPSS Statistics (version 22.0; Static Base and Advanced Statistics, IBM Japan, Tokyo, Japan).

## Results

Written informed consent to participate in the study and publish the results was initially obtained from 34 pregnant women. Of these, 18 women dropped out for the following reasons: already hospitalized for delivery (*n* = 9), suffering from a disorder or disease (*n* = 2), inconvenience of schedule (*n* = 4), unknown (*n* = 3). Finally, 16 low-risk pregnant women (mean age, 29 years; *SD*, 2.2) were evaluated. The participant flow diagram is shown in Fig 1.

### Characteristics of the participants

The demographic factors of the participants, outcomes in relation to the intervention period, and maternal and fetal outcomes are shown **Table 1**.

**Table 1:** Characteristics of the participants.

			(*n* = 16)
***Demographic factors***			
	Age (in years)		29 [2.2]
	Living with partner		16 (100)
	Married		16 (100)
	Full-time job		10 (62.5)
	Education≥ 12 years		12 (75.0)
	Primiparous		13 (81.3)
***Outcomes in relation to intervention period***			
	Gestational weeks		38.9 [2.5]
	Bishop score on day 1		2.8 [2.5]
	Bishop score on day 3		3.5 [2.4]
	Uterine hyperstimulation		0 (0.0)
	Severe variable deceleration		1 (6.3)
***Maternal and fetal outcomes***			
	Gestational weeks at delivery		39.8 [0.7]
	Onset of labor		
		Spontaneous	12 (75.0)
		Induced	4 (25.0)
	Onset of labor within 72 hours after intervention		6 (37.5)
	Mode of delivery		
		Vaginal delivery	15 (93.8)
		Instrumental delivery	0 (0.0)
		Cesarean section	1 (6.3)
	Apgar score at 5 min (mean)		9 [1.0]
	Apgar score at 5 min (< 7)		1 (6.3)
	Mean birth weight (grams)		2930 [200]
	NICU admission immediately after birth		1 (6.3)
	Meconium stained liquor (≥ 2+)		2 (12.5)
	Stillbirth		0 (0.0)

Values are expressed as mean [standard deviation] or number (percentage) of women.

### Salivary oxytocin

Among a total of 282 saliva samples, OT level was measured in 142 samples (missing rate: 49.6%). Of the 16 women who participated in the intervention, only 3 women could complete the collection of all 18 saliva samples.

**Fig 3:**
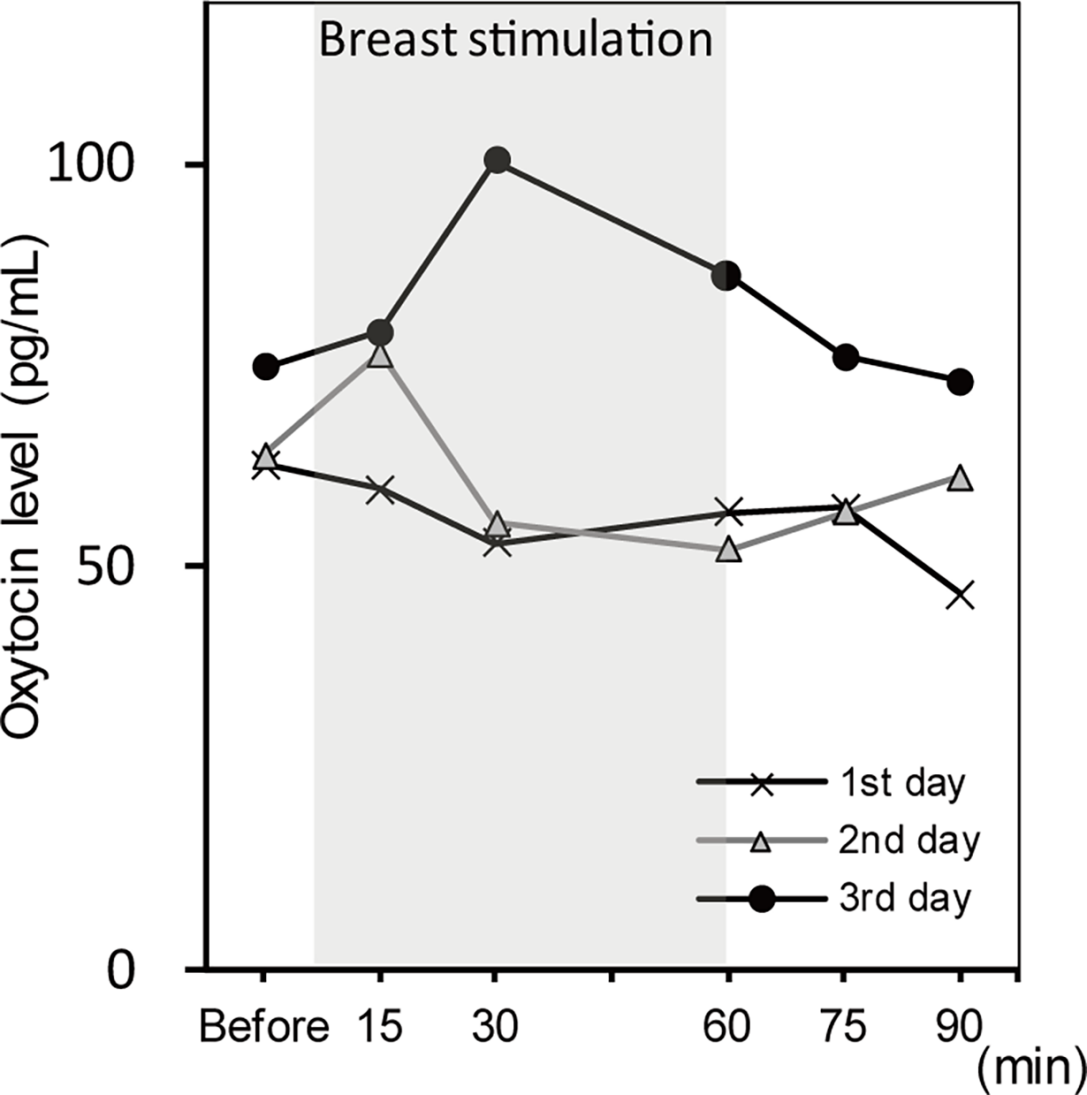
Median levels of salivary oxytocin on days 1–3.

After breast stimulation, the median level of salivary OT did not increase on day 1, increased only at 15 minutes on day 2. The highest level of salivary OT on day 3 was observed at 30 minutes after the breast stimulation (**Fig 3**). The median levels of salivary OT are described because the values have deviated from the normal distribution (**Table 2**).

**Table 2:** Salivary oxytocin levels (*n* = 16, 142 samples).

								*(pg/mL)*
Day			*n*	Min	Max	*M*	*SD*	Median
First	O_1_	Baseline	13	43.0	209.5	82.2	48.1	62.7
	O_2_	15 min	9	43.7	147.5	70.5	31.9	59.7
	O_3_	30 min	10	32.4	136.7	66.2	37.2	52.9
	O_4_	60 min	6	35.1	102.1	60.2	27.3	56.7
	O_5_	75 min	8	45.2	80.3	61.1	12.0	57.4
	O_6_	90 min	9	27.0	72.8	51.4	16.6	46.6
Second	O_1_	Baseline	7	25.6	248.7	101.9	89.3	63.8
	O_2_	15 min	7	42.1	139.3	75.9	35.7	76.5
	O_3_	30 min	8	30.5	123.6	74.4	38.7	55.4
	O_4_	60 min	7	43.3	134.4	75.4	37.0	52.2
	O_5_	75 min	5	36.5	96.9	62.6	25.9	56.8
	O_6_	90 min	9	31.2	188.2	79.5	56.6	61.2
Third	O_1_	Baseline	10	35.2	648.8	138.0	183.4	74.8
	O_2_	15 min	5	39.4	434.8	137.1	167.4	79.1
	O_3_	30 min	8	34.9	190.3	109.2	57.5	100.3
	O_4_	60 min	5	43.0	205.7	100.8	61.5	86.0
	O_5_	75 min	8	26.3	139.3	79.9	36.4	76.0
	O_6_	90 min	8	24.2	268.1	93.8	76.6	72.9

M, Mean; SD, Standard deviation

We used a linear mixed model with the OT level as the dependent variable and the day and time of intervention as the fixed effects. The OT level on the third day of intervention tended to be higher than that on the first day (*M* = -53.2, *SE* = 32.9, *p* = .111) (Data not shown). The OT level according to the time of day was the highest on the baseline and it decreased with time. We carried out multiple imputations and results of the 5 imputed datasets with mixed models methods indicating the mean of the pooled estimates are shown in **Table 3**.

**Table 3:** Mean estimates of salivary oxytocin level with mixed models methods after multiple imputations for missing data (*n* = 16, 288 samples).

							*(pg/mL)*
						95% CI	
Day			*n*	*M*	*SE*	Lower	Upper
First	O_1_	Baseline	16	81.9	12.5	57.5	106.3
	O_2_	15 min	16	71.0	12.4	46.6	95.4
	O_3_	30 min	16	66.6	12.5	42.1	91.1
	O_4_	60 min	16	60.3	12.5	35.8	84.8
	O_5_	75 min	16	60.7	12.4	36.3	85.1
	O_6_	90 min	16	51.1	12.5	26.7	75.6
Second	O_1_	Baseline	16	102.7	12.7	77.8	127.7
	O_2_	15 min	16	76.5	12.5	51.9	101.0
	O_3_	30 min	16	73.7	12.5	49.2	98.1
	O_4_	60 min	16	75.8	12.5	51.3	100.3
	O_5_	75 min	16	62.7	12.4	38.3	87.1
	O_6_	90 min	16	79.3	12.5	54.8	103.7
Third	O_1_	Baseline	16	137.4	12.7	112.5	162.4
	O_2_	15 min	16	139.4	12.8	114.3	164.4
	O_3_	30 min	16	110.1	12.6	85.4	134.8
	O_4_	60 min	16	100.7	12.5	76.2	125.2
	O_5_	75 min	16	79.5	12.5	55.0	104.0
	O_6_	90 min	16	93.9	12.6	69.3	118.5

M, Mean; SE, Standard error; CI, Confidence interval

**Fig 4:**
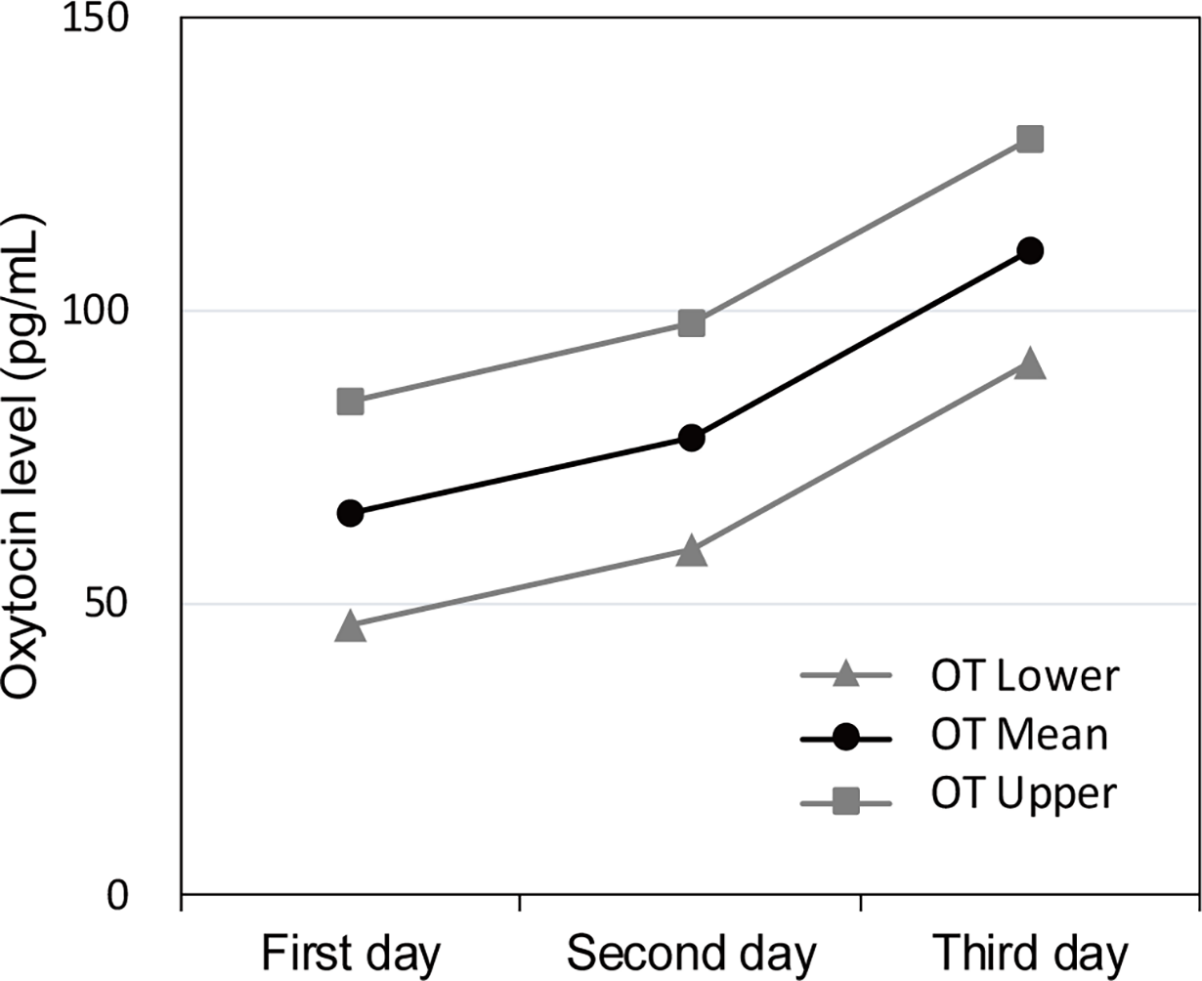
Mean estimate levels of salivary oxytocin and 95% confidence intervals for first, second, and third days with linear mixed models after multiple imputation.

When compared with the mean OT levels of the original data in **Table 2**, the estimated values in **Table 3** were nearly similar. Mixed models were performed to analyze the effects of breast stimulation on salivary OT level according to the day and time of intervention (**Table 4**). As a result, the OT level was significantly lower on the first day of intervention than on the third day of intervention (*M* = -55.5 pg/mL, *SE* = 19.5, *p* < .001). The mean estimates of the OT level according to the day of intervention using the mixed models are shown in **Fig 4**.

**Table 4:** Results of mixed models methods for assessing the effects of breast stimulation on salivary oxytocin level according to the day and time of intervention (*n* = 16, 288 samples).

									*(pg/mL)*
								95% CI	
			*n*	*M*	*SE*	*t-*value	*p-*value	Lower	Upper
Day	First		16	-55.5	19.5	-2.85	< .001	-93.8	-17.3
	Second		16	-34.7	19.0	-1.83	.067	-71.9	2.5
	Third		16	Reference					
Time	O_1_	Baseline	16	Reference					
	O_2_	15 min	16	1.9	12.7	0.15	.881	-23.3	27.1
	O_3_	30 min	16	-27.4	15.6	-1.75	.080	-58.0	3.3
	O_4_	60 min	16	-36.8	17.0	-2.17	.030	-70.0	-3.5
	O_5_	75 min	16	-57.9	17.8	-3.25	.001	-92.9	-23.0
	O_6_	90 min	16	-43.6	18.8	-2.32	.020	-80.4	-6.8

M, Mean; SE, Standard error; CI, Confidence interval

Values are expressed as mean ± *SD* between the OT receptor polymorphisms (**Table 5**). The GG carrier with rs53576 is known to be associated with a high risk for slow delivery. However, the present study showed a higher OT level before the intervention in the participants with the GG type.

**Table 5:** Participants with oxytocin receptor single nucleotide polymorphism (n = 13).

							*(pg/mL)*
SNP		*n*	Min	Max	*M*	*SD*	Median
rs53576	GG	3	58.7	209.5	109.9	86.2	61.6
	AG/AA	10	43.0	151.8	73.8	33.1	68.4
rs2254298	GG	7	43.3	209.5	79.7	58.2	61.6
	AG/AA	6	43.0	151.8	85.0	38.3	79.6
rs1042778	TT	0	-	-	-	-	-
	GG	10	43.3	209.5	87.8	53.2	68.4
	GT	3	43.0	85.2	63.3	21.1	61.6

SNP, Single nucleotide polymorphism; M, Mean; SD, Standard deviation

Thus, the Bishop score was confirmed not to show a significant change (**Table 1**). In addition, there was no correlation between the change in the Bishop score and the OT level on the third day.

### Maternal outcome

Previous studies have evaluated the number of pregnant women who had onset of labor within 72 hours after breast stimulation. In the present study, 6 (37.5%) of the 16 women had onset of labor within 72 hours after the intervention.

Adequate uterine contraction was obtained in 8 (50.0%) of 16 women on day 1, 6 (37.5%) of 16 women on day 2, and 5 (33.3%) of 15 women on day 3. This could not be confirmed in 4 women. In the induction of labor group (*n* = 4), 2 women who had labor induction for being beyond 24 hours after premature rupture of membranes (PROM) were confirmed to have adequate uterine contraction on 2 successive days. However, the other 2 women who had labor induction for being beyond their term were not confirmed to have adequate uterine contraction.

### Practicality of experimental methods

A total of 282 saliva samples were collected from the 16 participants. One woman had rupture in the night of day 2, thus the experimental intervention on day 3 was not implemented and 6 samples were not collected. Of these, 140 samples (49.6%) had insufficient amount of saliva for analysis of the OT level, making the final number of samples analyzed as 142. We collected 6 contiguous samples from 5 participants on day 1. Among them, we collected 18 contiguous samples from 3 participants for 3 days.

One woman showed non*-*reassuring fetal heart rate patterns after breast stimulation during the experiment. Severe variable deceleration for more than 2 minutes was observed after 45 minutes of breast stimulation on day 3, prompting the immediate discontinuance of breast stimulation. After 30 minutes, permission to restart the breast stimulation was obtained from the obstetrician. Thus, stimulation was performed for 15 minutes. The woman delivered without any problems for over a 4-day post-intervention.

There were 3 women (18.7%) with PROM. One had rupture in the night of day 2 with her labor starting after 10 minutes. As the other 2 women had no onset of labor after 24 hours from rupture, labor was induced in accordance with the hospital policy.

Ten days after the breast stimulation intervention, 1 mother was admitted for labor induction. Four days after the start of the labor induction, caesarian section was performed because of fetal distress. The delivered baby had meconium stained liquor and an Apgar score of < 7, and was admitted to the neonatal intensive care unit (NICU) immediately after birth (**Table 1**).

### Acceptability of experimental method

The dropout rate after agreeing to participate was 53%. The most common reason for dropping out was their already being hospitalized for delivery. There was no participant who voluntarily dropped out after starting the intervention.

The highest level of fatigue from the saliva collection procedure as indicated by the VAS score was observed on day 3. The average time of saliva collection was 4.4 minutes (*SD*, 2.0). The degree of pain from breast stimulation remained almost the same, but the level of fatigue from breast stimulation decreased daily.

Most women (75%) were not uncomfortable with touching their nipple. All women responded that the instructions for breast stimulation were easy to understand. More than half of the total number of women answered that the rhythm and strength of the breast stimulation were appropriate. All the participants hoped to perform this protocol in their next pregnancy, as well as recommend this method to their friends.

This breast stimulation method can be continued at home in the future by the women themselves by watching a video to confirm the stimulation method. About 90% of the women answered that the most suitable environment for continuing this method was while watching the television.

## Discussion

### Changes in oxytocin level by breast stimulation

In this study, the median OT level was highest on day 3, particularly 30 minutes after the intervention. There were slight increases in the OT level on days 1 and 2, although the changes were not distinct. This result resembles that of a previous report which investigated OT level 1 day after breast stimulation [18]. Although several days were needed to perform the procedure to promote the spontaneous onset of labor [8], previous studies used only a 1-day procedure.

To the best of our knowledge, the present study is the first to investigate the physiological background of long hours of breast stimulation, which has been reported to be effective in promoting the spontaneous onset of labor. Hence, the changes in salivary OT level induced by breast stimulation may possibly indicate the effect of repeat stimulation. Prevost et al. reported that 70% of women on the third trimester of pregnancy had a higher plasma OT level than women on the first trimester of pregnancy [35]. Thus, the increase in the OT level in the present study may be a physiological phenomenon. Alternatively, this occurred owing to the positive feedback by breast stimulation. However, to the best of our knowledge, there is as yet no short-term study describing changes in the OT level by breast stimulation with time. Future experiments with a control group are warranted.

### Onset of labor within 72 hours after intervention

Of the 16 women who participated in this study, 6 women had onset of labor within 72 hours after the intervention. In previous studies, women who had onset of labor within 3 days accounted for 33% to 36% of the intervention group [10,14]. The same trends of rates are shown in this report.

In terms of the Bishop score, there was no significant difference before and after the intervention. A previous study using the same procedure reported an increase of 3.9 points as a change in the Bishop score in the intervention group compared with the control group [10]. The average age of the subjects was 23 years and the intervention was performed around the expected date of confinement. This may account for the difference in the results. In other previous studies that included more than 100 women, long hours of stimulation [14] and long-term stimulation [15] resulted in an increase in the Bishop score. This preliminary study which involved a small sample size showed no effect on the Bishop score.

### Practicality of experimental methods

We collected 1.0 mL of saliva for duplicate assays. However, this amount may have been insufficient for the assays because of the possible large amount of mucin. Therefore, it is considered that the required minimum amount of saliva to be collected should be 1.5 mL. A limitation of this study is that missing data were most likely inevitable because of the insufficient amount of saliva collected for OT level measurement. This situation implies that to be able to collect more saliva, the participants should brush after meals and rinse their mouth before the intervention. As water was taken only once, more saliva could have been collected if water was taken before each collection procedure.

The reason for the many high baseline salivary OT levels in the present study is not clear. Previously, the highest baseline salivary OT level in lactating women was reportedly observed before breastfeeding [25]. However, a low baseline level was also reported after breast stimulation in pregnant women [17]. Importantly, the experimental environment is crucial for the rigorous control of external factors that affect baseline OT levels. Thus, adequate rest time should be considered before the collection of baseline samples.

Fetal heart rate deceleration was observed in this study after breast stimulation. However, the subsequent stimulation performed after obtaining permission from the obstetrician resulted in a normal delivery. Taken together, breast stimulation as a method of promoting the spontaneous onset of labor is safe in low-risk pregnant women.

### Acceptability of experimental method

The degree of burden of the experimental method in terms of saliva collection and breast stimulation showed a moderate score, indicating its acceptability among the participants. Most of the women were not uncomfortable with the protocol, thus breast stimulation may be considered acceptable among Japanese pregnant women.

The limitations of this preliminary study were the absence of a control group and the limited number of participants. In future studies, a large sample size and inclusion of a control group are warranted to clarify in more detail the specific effects of the breast stimulation intervention used.

## Conclusion

This study investigated the physiological background of long hours of breast stimulation in relation to labor induction. Following a 3-day breast self-stimulation intervention protocol for the induction of spontaneous onset of labor in low-risk pregnant women, the mean OT level showed the highest values on day 3. The breast stimulation intervention approach used showed good feasibility in terms of practicality and acceptability among the pregnant women.

## Supporting information

S1 Table TREND statement checklist. (PDF)

S1 Text Original study protocol. (PDF)

S2 Text Study protocol in English. (PDF)
