# Supplementary material for: Effects of breast stimulation for spontaneous onset of labor on salivary oxytocin levels in low-risk pregnant women: A feasibility study
Source: PLoS One. 2018 Feb 15;13(2):e0192757. doi: 10.1371/journal.pone.0192757 (PMC5813971; doi:10.1371/journal.pone.0192757)
Supplement: S1 Text — (PDF) [file pone.0192757.s002.pdf]

2015 年 5 月 18 日

2015 年度聖路加国際大学大学院 研究計画書

ローリスク妊婦における陣痛発来を目的とした

乳房刺激による唾液中オキシトシンの変化：パイロットスタディ

Change Oxytocin in Saliva by Breast Stimulation for
Spontaneous Onset of Labor in Low Risk Pregnant Women: Pilot Study

高畑　香織

第1章　序論

I. 研究の背景

妊婦の 10%は妊娠 42 週以降まで妊娠が継続すると言われている(Olesen 2003; Roos 2010)。また、デンマークの Olesen ら(2003)は、正期産での周産期死亡は 0.3%だが、妊娠 42 週以降では 0.4%と報告している。過期産による有害事象を予防するために、一般的に薬剤による分娩誘発（以下、分娩誘発）が行われるが、自然な陣痛発来に比べると、その後の帝王切開が有意に増加するとされる（Kiesewetter et al, 2012)。その中でも特に、初産婦において、有意に分娩誘発後の帝王切開の増加が指摘されている（Henderson et al., 2013; Luthy et al., 2004; Yeast et al.,1999)。

現在補完代替療法によって自然に陣痛発来させる方法で効果があると考えられているのは、乳房刺激と卵膜剥離である（Kavanagh et al., 2005; Boulvain et al., 2005)。しかし、卵膜剥離は医療者が主体となる方法であり、実施時には著しい不快感や疼痛が生じる。一方、乳房刺激は妊婦自身で実施でき、安価であることから、これまで多く研究が行われてきた。Kavanagh ら（2005）は、719 名の妊婦を含む 6 試験の RCT についてシステマティックレビューを行った。その結果乳房刺激を行うと、72 時間以内に陣痛発来に至らない女性が減り（RR 0.67, 95% CI[0.60, 0.74]）、NNT （Number Need to Treat）は 4 という少ない人数で介入による効果が得られるとした。しかし、いずれの研究も 20 年以上前に実施され、無作為化の手法など不明な点も多い。また、各研究の介入時間や期間および実施方法は統一されておらず、妊婦自身が指で刺激する方法や、電動搾乳器を使用する研究も含まれる。高畑（2014）が実施した本邦の実態調査では、分娩前 1 週間において陣痛を起こすことを目的に乳房刺激を行ったのは 530 名中 93 名であったが、先行研究で有効とされる 3 日間合計 3 時間以上の乳房刺激を実施していたのはわずか 2 名であった。

陣痛が発来する機序は諸説あり、複数の要因が関係していると考えられている。乳房刺激による自然な陣痛発来を導くメカニズムとして、オキシトシン（以下、OT とする）の関与が考えられている。OT は個人間での濃度の違いが大きいホルモンであり、ストレスや不安、社会行動と密接な関連があると考

えられている（尾仲, 2014)。Fuchs et al(1991)によって、妊娠末期・分娩第1期・分娩第2～3期までにおいて、血中OTの濃度およびパルスの振幅および頻度のいずれもが増加していることが明らかにされている。Contraction Stress Test（以下、CSTとする）でオキシトシン点滴静脈注射を使用する代わりに乳房刺激を実施していた歴史から、1980年代に乳房刺激と血中OT値の関連について研究した文献が散見される。CSTとは、子宮収縮に対する胎児心拍数の反応をみる検査である（池ノ上ら,2004)。主にハイリスク妊婦に対して、胎児予備能の評価を行うために、妊娠末期に実施されていた。Amico et al（1986）は、妊娠期における数分間の乳房刺激により血中OT値が上昇したと報告した。一方、Ross et al(1986)は、乳房刺激によるCSTによって、20名中9名で子宮収縮が生じたが、血中OT値は変化しなかったと報告している。これらの原因として、血中OTがパルス状に分泌されていること、パルスの頻度や振幅に個人差があること(Fuchs et al., 1991)の影響があると考えられる。さらに、OTの感受性には子宮筋層のオキシトシン受容体（以下、OTRとする）の関与が指摘されている。OTRは、妊娠後期から分娩開始までに50倍以上も増加する（Fuchs,1982;Kimura, 1996)。OTRが多いとOTに対する子宮の応答性は増加し、低濃度のOTでも子宮収縮の増強作用は強くなる（Cunningham et al., 2014)。しかし、OT受容体遺伝子多型によって、OTの感受性が低下することが指摘されている(Terkawi et al., 2012)。

OTは、唾液、尿、脳脊髄液、羊水などから採取することが可能である。唾液中のOT測定は、他の測定手法と比べて被験者への侵襲度が低く、簡便なOT測定方法として近年注目されている。また、唾液中OTは血中OTのパルスの影響を受けにくい値が得られると考えられる。採取した唾液を濃縮した後にenzyme immunoassayにて分析キットの推奨する分析方法に則って測定することでより安定した測定値が得られるとされる(McCullough et al., 2013)。

以上より、自然な陣痛発来を目的とした乳房刺激は、その有効性として子宮頸管の熟化や72時間以内の陣痛発来が確認されている。また、その根拠となるRCTの無作為化の手法には疑問点が多く、最も有効な介入時間は不明である。さらに、OTR遺伝子多型の影響は考慮されていない。今後、妊娠期のセルフケアとして取り入れ可能な陣痛発来を目的とした乳房刺激の方法を検討する

ために、乳房刺激による唾液中 OT への影響を分析することにした。

II. 研究の目的

ローリスク妊婦における妊娠期の陣痛発来を目的とした乳房刺激の方法を検討する本研究に先立ち、下記について検討する。

1. 乳房刺激による下記項目の関連について分析する

   プライマリーアウトカム

   ① 唾液中オキシトシンの経時的変化

   セカンダリーアウトカム

   ②著明な子宮収縮、③頸管熟化度、④介入後 72 時間以内の自然陣痛発来
2. 妊婦の個別的な性質が唾液中オキシトシンおよび分娩アウトカムに影響しているかを、①オキシトシン受容体遺伝子多型、②不安尺度、③養育環境、④デモグラフィックデータによって分析する
3. 乳房刺激および唾液採取など研究手順の実行可能性について検討する

III. 研究の意義

本研究により得られる成果・発展性については以下が期待される。

1. 唾液中オキシトシンとオキシトシン受容体遺伝子型の影響の傾向を知ることによって、陣痛発来のための乳房刺激の最も効果的な介入時間の検討への示唆を得ることができる
2. 将来的に、対照群を持つ 2 群比較あるいは RCT にて乳房刺激の有効性が確認されるならば、過期産の予防、薬剤等による分娩誘発を減少させるための有効な補完代替療法へつながる可能性がある

IV. 用語の操作的定義

乳房刺激：手指や器具を使用して、乳頭を含む乳房を刺激すること。

自然な陣痛発来：薬剤や器械等を使用せずに分娩へ至る陣痛が起こること。

自然分娩：薬剤や器械等を使用せずに経腟分娩に至ること。

頸管熟化度（BS）：0～3 点となる 5 項目のスコアで、13 点が満点となる。点数が高いほど頸管は熟化していることを示す。

著明な子宮収縮：1回40秒以上続く子宮収縮を10分間に3回以上認めること。

第2章　研究方法

I. 研究デザイン

本研究は、一群時系列分断デザインによる準実験研究である。

II. 研究の対象

1. 研究対象者

研究の対象は、以下の条件をすべて満たした女性とする。

(1) 年齢25歳以上35歳以下

(2) 単胎、頭位で経腟分娩を予定している者

(3) 妊娠週数38週0日以上40週6日未満

(4) 日本語の読み書きができるアジア人女性

(5) 主治医または担当助産師によって研究参加の了承が得られる者

上記のうち、以下のいずれかに該当する女性は除外とする。

(1) 医師の処方による内服がある者（ただし便秘薬・鉄剤を除く）

(2) 医学的・産科的合併症がある者（妊娠高血圧症候群、妊娠糖尿病、内分泌疾患、子宮形態異常など）

(3) 精神疾患既往歴のある者

(4) 生殖補助医療による不妊治療歴（一般不妊治療を除く）のある者

(5) 非妊娠時BMI25以上の者

(6) 陣痛誘発を予定している者

(7) 過期産の既往のある者

(8) 帝王切開術の既往のある者

(9) 授乳中の者

2. 研究対象施設および対象者数

分娩取り扱いがあり、入院施設のある病院一施設にて実施する。研究対象者数は 10～15 名程度とする。

3. 施設のリクルート方法

研究協力施設に対し、施設への依頼文書(資料 1)および施設用調査手順書（資料 2）を用いて、研究協力の依頼を行う。研究協力の承諾が得られた場合、研究協力施設内にて、本研究に関する情報公開としてポスター(資料 10)で本研究の趣旨に関する掲示を行う。

4. 対象者のリクルート方法

産科外来のカルテからリクルート対象の妊婦を抽出し､対象妊婦が妊娠 34 週以降に定期健康診査へ訪れた際に対象者への依頼文書（資料 3）およびリーフレット（資料 4）にて調査目的および内容を説明する。同意を得られた妊婦を対象とする。

III. 調査期間

2015 年 5 月から行う。

IV. 調査手順と方法

介入期間は 3 日間を設定し､1 日の乳房刺激の時間は左右 15 分ずつ合計 1 時間実施とする。刺激は､妊婦自身が自分の指で行う。刺激の標準化を図るため､膣圧測定器にて刺激の強さと電子メトロノームにて刺激テンポの目安を示す。胸部の露出が容易な服装を着用してもらい、乳房刺激の際は指定した授乳用ケープを用いる。付き添い者の同室は許可しない。研究全体の流れ（図 1）および介入期間の流れ（図 2）を示す。

1. 介入 1 日目

1） 来院後、研究協力者 1 名にて内診台を使用して内診し、Bishop Score の測定を行う
2） プライバシーを保てる部屋で、当日の流れについて説明する
3） 質問紙 1 プレテスト（資料 7）を記入してもらう。唾液摂取 30 分前のた

め、水分摂取終了とする

4) 綿棒を使用し口腔粘膜を採取する。

5) 20 分間休憩をする。ファーラー位にて NST モニターを装着してから体温を測定する。

6) 休憩中に、研究者または研究協力者が乳房刺激の方法を説明する。膣圧測定器（OWMED 社製 MizCure）を使用して、刺激圧（mmHg）の目安を示す。妊婦にも実施してもらう

7) 乳房刺激の方法は、15 分間ずつ交互に、右乳房から手指で乳頭・乳輪部のマッサージを行う。刺激は、主に拇指・示指・中指の 3 本を使用し、正面から、乳輪部の外側から乳頭までつまみ圧迫する動き、把持したまま回転する動き、指つまみや側方つまみの動きを適宜組み合わせる。適宜左右の手を交換しながら刺激を行う。潤滑剤（馬油）は、研究者が用意し、1 日ですべて使いきってもらう。電子メトロノームを使用し、1 分間に 70 回程度の刺激を実施する。メトロノームの音は、対象者が聞こえる範囲で最も小さい音量設定にする。対象者が持続的なメトロノームの使用に不快感を訴えた場合は、刺激のテンポを確認するのは 15 分毎とする。

8) 1 回目の唾液採取を行う（刺激前値）

9) 研究者が用意した DVD の視聴を開始する

10) 右乳房刺激を 15 分実施した後、2 回目の唾液採取を行う（刺激後 15 分値）

11) 左乳房刺激を 15 分実施した後、3 回目の唾液採取を行う唾液採取直後に、水分摂取をしてもらう。（刺激後 30 分値）

12) 右乳房刺激を 15 分実施する

13) 左乳房刺激を 15 分実施した後、4 回目の唾液採取を行う（刺激後 60 分値）

14) 乳房刺激終了 15 分後、5 回目の唾液採取を行う（刺激後 75 分値）

15) 乳房刺激終了 30 分後、6 回目の唾液採取を行う（刺激後 90 分値）

16) モニターは、乳房刺激終了後 30 分間まで装着し、胎児が reassuring であることの確認を行ってから終了する

17) 前駆陣痛が生じた場合、対象者と相談し、院内に留まるか帰宅するかを

検討する。子宮収縮があっても、自覚が弱い場合は帰宅可とする。本陣痛であると判断した場合およびその判断ができない場合は、主治医または研究担当看護職者へ報告し、指示を仰ぐ。

18) 6回目の唾液採取後に、乳房刺激による疲労と疼痛についての質問紙 2 プレテスト①（資料 8）を実施する。

2. 介入 2 日目

1) 来院後、プライバシーを保てる部屋で当日の流れについて説明する

2) 介入 1 日目と 5)から 18) まで同様に実施する。

3. 介入 3 日目

1) 来院後、プライバシーを保てる部屋で当日の流れについて説明する

2) 介入 1 日目と 5)から 17) まで同様に実施する。

3) 6回目の唾液採取後に質問紙 3 プレテスト②（資料 9）の記入を実施する

4) 内診台を使用して Bishop Score の測定を行う。

＜研究全体の流れ＞ 図1

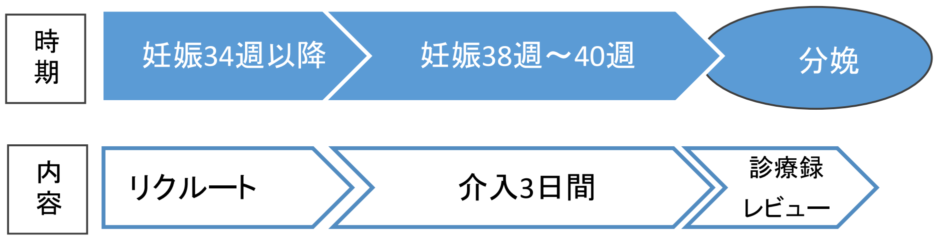

＜介入期間の流れ＞

介入1日目

経過時刻	0:00	0:15	0:30	0:45	1:00	1:15	1:30	1:45	2:00	2:15	2:30	2:45	3:00
	来院<br>トイレ	質問紙記入<br>（資料7）		20分休憩　検温<br>口腔粘膜採取								質問紙記入<br>（資料8）	
乳房刺激<br>（片側15分間ずつ）						●<br>右	左	右	左 →				
胎児心拍ﾓﾆﾀﾘﾝｸﾞ<br>唾液採取<br>Bishop　score測定	BS			●	①		②	③		④	⑤ →	⑥	

介入2日目

経過時刻	0:00	0:15	0:30	0:45	1:00	1:15	1:30	1:45	2:00	2:15	2:30	2:45	3:00
	来院<br>トイレ	20分休憩	検温							質問紙記入<br>（資料8）		帰宅	
乳房刺激<br>（片側15分間ずつ）				●<br>右	左	右	左 →						
胎児心拍ﾓﾆﾀﾘﾝｸﾞ<br>唾液採取		●	①		②	③		④	⑤ →	⑥			

介入3日目

経過時刻	0:00	0:15	0:30	0:45	1:00	1:15	1:30	1:45	2:00	2:15	2:30	2:45	3:00
	来院<br>トイレ	20分休憩	検温							質問紙記入<br>（資料9）		帰宅	
乳房刺激<br>（片側15分間ずつ）				●<br>右	左	右	左 →						
胎児心拍ﾓﾆﾀﾘﾝｸﾞ<br>唾液採取<br>Bishop　score測定		●	①		②	③		④	⑤ →	⑥	BS		

図 2

V. 測定用具とデータ収集内容

プライマリーアウトカム

1. 唾液中オキシトシン値

1) 測定具と解析

唾液採取には 2.0ml エッペンチューブとストローを使用する。オキシトシン値測定は、長崎大学大学院医歯薬学総合研究科篠原一之教授研究室にて、ELISA 法による解析を行う。

2) 測定条件

唾液中オキシトシン値は、飲食や活動など、さまざまな要因の影響を受けることが考えられるため、条件をそろえて測定することが望ましいと考えられる。従って、条件として以下を設定する。

① 研究参加前日は性交・アルコール摂取は禁止する
② 研究参加当日は、朝食を摂取する
③ 採取時間は 10 時から 15 時の間とする
④ 採取 1 時間前より喫煙、歯磨き、飲食禁止（水のみ摂取可）
⑤ 採取 30 分前までに水の摂取を終える
⑥ 平熱である
⑦ 口紅の使用禁止

3) 測定環境

研究期間中、研究のために協力施設内に設置された測定専用スペースを使用する。スペースは、プライバシーが保てるように配慮する。

4) 測定方法

本研究では、ストローによる唾液採取（Passive Drool）を行う。

① 顔を前に少し倒して口を閉じ、唾液が自然に口にたまるのを 1～3 分間待つ。チューブには、対象者の ID、採取日時および測定回数を記載する。

② 3 分経過後、ストローを使って、1.0 のメモリのところまでエッペンチューブの中に唾液を流す。このとき唾液が泡沫状にならいように注意する。

③ 唾液がたまらない場合はさらに数分間、口を閉じて待つ

④ 唾液がたまったら、ストローを捨てて、カチッと音がなるまでしっかり蓋をしめる。使用済みストローは処分する。

⑤ 研究者または研究協力者が、保冷剤の入った保冷庫にて保存する

⑥ -80 度の冷蔵庫にて検体を冷凍する

⑦ 一定数検体が集まった時点で、長崎大学へ検体を郵送する

セカンダリーアウトカム

2. オキシトシン受容体遺伝子多型

1) 測定具と解析

OTR 遺伝子多型の分析のため、口腔粘膜を採取する。使用物品は、口腔粘膜採取用綿棒（以下、綿棒)、1.5 ml チューブ、ジップ付きナイロン袋を各 1 セットである。聖路加共同研究ラボラトリの周尾卓也主任研究員の指導の下、解析を行う。

2) 測定条件・環境

測定条件は、食後 1 時間以上経過していることとする。他者の遺伝子混入を防ぐため、綿棒の先に触れることや、異物混入を防ぐよう注意を行う。

3) 測定方法

(1) ジップ付きナイロン袋へ採取日と ID を記載しておく

(2) 綿棒のビニールを片方だけ取り外す。

(3) 綿棒の先端を口の奥に入れ、右頬の口腔粘膜を普通の力で幅広く、30 秒（30 回）程度、押し当てて擦る。

(4) 綿棒の先ほどと同じ部分で、左頬の口腔粘膜も同様に採取する。

(5) 擦った先をチューブに差し込み、綿棒を容器に収める。

(6) チューブのフタを開けたまま、風通しのよい冷暗所で 2 時間以上、自然乾

燥させる。

(7) 乾燥後ジップ付きナイロン袋に入れ、密封する。

3. 質問紙

1) 不安尺度：状態特性不安検査(STAI；State-Trait Anxiety Inventory)

STAI は Spielburger ら（1970）によって作成された尺度であり、不安を状態不安と特性不安とに分ける。状態不安は自律神経の興奮などを伴う一時的、状況的な不安状態を示し、「現在、今どの程度感じているか」を回答させる。特性不安は、ストレス状況に対して状態不安を喚起させやすい傾向である比較的安定した個人内特性と捉えられ、「ふだん、一般にどの程度の状態か」を回答させる。STAI は周産期の女性の不安を測定する尺度としての妥当性が確認されている（Meades et al.,2011)。また、産褥期の女性で、血漿中 OT と STAI には中等度の逆相関が認められている（Alison et al., 2013)。今回の研究では、カットオフ値を設け、不安の強さによる唾液中 OT 分泌との関連を分析するために用いる。

STAI は、清水ら(1981)によって日本語版が作成され、妥当性と信頼性が確認されている。本研究では、清水ら（1981）による日本語版 STAI を用い、状態不安・特性不安に対して 20 項目を「全くそうでない」から「全くそうである」までの 4 段階リッカート尺度で実施する。

2) 乳房刺激の介入方法の検討に関する質問紙

陣痛発来のための乳房刺激の介入方法を検討するために、介入終了後の対象者に、研究者が作成した質問紙に回答してもらう。

介入 1 日目と 2 日目の終了後に行う質問紙 2 ポストテスト①（資料 8）は同じものであり、乳房刺激や唾液採取の疲労感などに対して、Visual analog scale（以下、VAS とする）を使用して回答してもらう。質問用紙は 1 枚で、3 分程度要する。

介入 3 日目の終了後には、質問紙 3 ポストテスト②（資料 9）を行う。質問用紙は 2 枚であり、5 分程度を要する。ポストテスト①と同様に、乳房刺激や唾液採取の疲労感などに対して、VAS を使用し、刺激の強さやテンポの質問に

対しては、5 段階リッカート尺度を用いた。また、不快感、指導のわかりやすさ、次回妊娠時も実施したいか、友人にも情報提供したいと思うかについては、「大変そう思う」から「そう思わない」まで、4 段階リッカート尺度を用いた。そのほかに、介入を実施する環境や刺激方法などについても質問する。

3.対象の基礎データ

データ収集は作成した質問紙を用いる。収集内容は、年齢、身長、体重、非妊娠時体重、非妊時 BMI、産科歴、過去の陣痛の状況、分娩予定日、妊娠週数、実母が過期産であったか、葉酸の摂取歴、服薬歴、職業、産前休暇取得し始めた妊娠週数、婚姻状態、パートナーとの同居、学歴、世帯年収である。

4. 介入によるデータ

Bishop Score による頸管熟化の評価。Bishop Score は、内診法であり、評価者により誤差が生じやすいため、研究協力者 1 名（研究者以外）で評価をする。また、胎児心拍モニターを使用し、過剰子宮収縮の有無、胎児機能不全の有無を観察する。介入 2 日目、3 日目の来院後に、介入終了後の自覚的子宮収縮を確認する。

5. 分娩アウトカム

分娩に関するデータは、対象の分娩終了後、診療録より収集する。収集内容は、72 時間以内の自然陣痛発来有無、陣痛誘発／促進、分娩週数、分娩様式、分娩異常の有無と内容、分娩所要時間、分娩時出血量、出生時の児体重、児の性別、アプガースコア（1 分および 5 分)、羊水混濁の有無と程度、新生児搬送／NICU への入室である。

6. データ分析方法

各変数の基本統計量を算出する。変数間の関連や差を分析するために χ^2 乗検定、t 検定を用いる。分析には、統計ソフト IBM SPSS Statistics version 20.0 for Windows を使用し、両側検定 $p<0.05$ にて有意とする。また、すべての研究過程を通してデータの収集や分析は、母性・助産学領域の専門家、統計学の専

門家のスーパーバイズを受ける。

尺度の欠損値は平均値で補完する。その他の欠損値は、そのまま欠損として取り扱う。測定検体の異常により、検査値の信頼性が得られない場合は、欠損データとして取り扱う。

分析の内容は主に以下の点である。

1) 対象者のベースラインの分析（年齢、産科歴、学歴、世帯収入、BMI、介入時の妊娠週数）
2) 測定ポイント毎の唾液中オキシトシン値の平均値、中央値、範囲等の算出（個人内、個人間での絶対値の推移）
3) 介入前後のデータの比較（介入前後の Bishop Score、著明な子宮収縮の有無、過剰子宮収縮の有無、著明な徐脈の有無）
4) 母体のアウトカムについて平均値、中央値、範囲等の算出（分娩週数、分娩開始方法、分娩様式、分娩所要時間、羊水混濁、分娩時出血量）
5) 児のアウトカムについて平均値、中央値、範囲等の算出（アプガースコア、出生時体重、胎児機能不全、新生児搬送／NICU への入室）
6) 唾液中オキシトシン値と各項目間（介入後 72 時間以内の陣痛発来、頸管熟化度、著明な子宮収縮、その他アウトカム）の関連
7) オキシトシン受容体遺伝子型と各項目間（唾液中オキシトシン値、介入後 72 時間以内の陣痛発来、頸管熟化度、著明な子宮収縮、その他アウトカム）の関連
8) 日本語版 STAI と唾液中オキシトシン値、分娩アウトカムの関連

VI. 倫理的配慮

1. 介入研究における対象者の個人の人権擁護

「人を対象とした医学系研究に関する倫理指針」に従い、本研究計画書を遵守して人権擁護に配慮して実施する。

1)対象者から同意を取得する方法

研究者または研究協力者は、対象者に対してあらかじめ倫理委員会の承認

が得られた同意説明文書等を用いて本研究の内容を十分に説明する。対象者には質問する機会および本研究に参加するか否かを判断するのに十分な時間を与え、本研究の内容をよく理解したことを確認したうえで研究開始前に本研究への参加について対象者本人の自由意思による同意を文書で得る（資料5)。同意書には、説明を行った研究者または研究協力者および同意した対象者が署名及び日付を記入し、原本は研究者が保管し、同意書(写)は対象者に渡す。

対象者の同意に影響を及ぼすような研究計画書の変更が行われるときは、速やかに対象者に情報提供し、研究に参加するか否かについて対象者の意思を確認するとともに、倫理委員会の承認を得て同意説明文書等の改訂を行い、対象者に再同意を得る。

2)対象者へもたらされる利益

研究に参加した対象者には、謝金として 3 日間で 9000 円(1 日あたり 3000 円）を口座振込みにて支払う。

3)研究協力によって生じる危険・不利益とそれに対する配慮

本研究で予想される有害事象は、乳房刺激実施による前期破水あるいは子宮の過剰収縮、およびそれに付随する胎児機能不全が考えられる。また、潤滑剤を使用した乳房刺激の実施により、アレルギー症状やかゆみなどの皮膚症状が発生する可能性が考えられる。

介入に際しては胎児心拍モニタリングを行い、本研究の実施中に有害事象を認めた際は、ただちに主治医に報告し適切な処置を行うとともに記録する。

4)事故が起きた場合の治療ならびに補填

本研究の実施に起因して有害事象が発生し、対象者に健康被害が生じた時は、適切な治療その他必要な措置を受けることができるように研究責任者、実施医療機関が対応する。提供される治療等には健康保険を適用し、その他金銭での補償は行わない。ただし、潤滑剤を使用した乳房刺激の実施によるアレルギー症状やかゆみなどの皮膚症状に対しては、研究者の補償保険にて

補填する。

5)研究対象者への任意性の確保

本研究への参加は対象者の自由意思により決定される。また一旦同意した後であっても、いつでもそれを撤回することが出来る。研究への不参加によってなんら不利益をもたらすことがない旨を説明する。研究参加への同意は同意書への署名によって確認し、同意書を受けとる際には、撤回書（資料 6）もお渡しする。

6)研究協力により発生する費用の有無とその負担者

本研究参加のために追加となる費用として、協力施設へ来院するための交通費がかかる可能性がある。この交通費は対象者への謝金に含まれているものとする。その他、本研究のために必要となる検査費用は研究者の研究費でまかなう。したがって本研究に参加することで対象者の医療費負担が増えることはない。その他、通常の診療で行う検査費用、診察代、薬剤費は対象者の保険診療で行われる。

2. 個人情報の保護

1) 本研究にかかわる個人データは全て登録番号にて匿名化して取り扱い、本研究の目的以外には使用しない。研究者はカルテ番号、氏名および生年月日と施設にて設定した研究対象者識別コードの対応表を作成する。データの管理は研究者が行うが、対応表は研究補助者が管理を行う。
2) 本研究結果は学術誌等で公表予定である。学会、論文等外部に発表する際は個人が特定されないよう匿名化する。また解析の段階でパソコンを使用する際には研究者本人のみがアクセスできるようパスワードの管理を徹底する。研究結果の開示を求める場合、研究協力の説明書に記載された研究者の連絡先に連絡をとることにより、開示が可能である。
3) 本研究で集めたデータ（データ収集用紙、フラッシュメモリー等）は、データ収集終了後より 5 年間保存した後、一切のデータを復元不可能な状態に消去、またはシュレッダーなどで細かく裁断し破棄する。

4) 予備研究終了後の本研究において、介入方法に大きな変更がない場合は、本調査で得られたデータを本研究に加える、データの二次利用を計画している。そのため、対象者へもこの点について説明を行い、同意を得る。また、二次利用研究を行う際は改めて研究倫理審査委員会へ申請し、承認された場合のみ実施する。
5) 研究協力者を雇用する場合は、研究で知り得る情報について、許可なく如何なる方法をもってしても、開示、漏洩もしくは使用しないことを誓約することへの同意を文書（資料 11）で得る。
