# Supplementary material for: Effects of breast stimulation for spontaneous onset of labor on salivary oxytocin levels in low-risk pregnant women: A feasibility study
Source: PLoS One. 2018 Feb 15;13(2):e0192757. doi: 10.1371/journal.pone.0192757 (PMC5813971; doi:10.1371/journal.pone.0192757)
Supplement: S2 Text — (PDF) [file pone.0192757.s003.pdf]

St. Luke’s International University, Graduate School

2015 Research Proposal

ローリスク妊婦における陣痛発来を目的とした

乳房刺激による唾液中オキシトシンの変化：パイロットスタディ

Change Oxytocin in Saliva by Breast Stimulation for Spontaneous Onset of Labor in Low Risk Pregnant Women: Pilot Study

Kaori Takahata

Introduction

Background

It is said that 10% of pregnant women continue pregnancy until after 42 weeks of pregnancy (Olesen 2003; Roos 2010). In addition, Olesen et al. (2003) in Denmark reported 0.3% perinatal mortality in the primary term but 0.4% after 42 weeks gestation. In general, medically induce labor is carried out to prevent adverse events due to postterm, but it is said that the subsequent caesarean section will be significantly increased as compared with the arrival of natural labor (Kiesewetter et al., 2012). Among them, especially in primiparas, there is a significant increase in caesarean section after induction of labor (Henderson et al., 2013; Luthy et al., 2004; Yeast et al., 1999).

Breast stimulation and membrane sweeping are considered to be effective in a method of naturally causing labor by present complementary replacement therapy (Kavanagh et al., 2005; Boulvain et al., 2005). However, membrane sweeping is a method that is mainly performed by medical personnel, and when it is performed, there is a remarkable discomfort and pain. On the other hand, a pregnant woman can perform her own breast stimulation, and since it is inexpensive, much research has been done so far.

Kavanagh et al. (2005) conducted a systematic review of six trials of RCT including 719 pregnant women. As a result, breast stimulation reduced the number of women who did not have labor within 72 hours (RR 0.67, 95% CI [0.60, 0.74]). However, all studies were conducted more than 20 years ago, and there are many unknown points such as randomization method. In addition, the time and duration of the intervention and the method of implementation of each study are not unified, including the method by which the pregnant women stimulated their nipples with fingers, and the method using electric powered breast pumps. In Takahata's (2016) survey conducted in Japan, 93 out of 530 women stimulated breast for the purpose of causing labor in one week before parturition but there were only two women who had breast stimulation for more than three hours total for three days.

There are various theories of the mechanism by which labor occurs, and it is thought that several factors are involved. Oxytocin (OT) is considered to be involved as a mechanism that leads to natural labor resulting from breast stimulation. OT is a hormone with a large difference in concentration among individuals and researchers think it is closely related to stress, anxiety, social behavior (Onaka, 2014). It has been revealed by Fuchs et al. (1991) that both plasma OT level and pulse amplitude and frequency are

increasing toward the end of pregnancy, the first trimester parturition, and second to third term of parturition. From the history that breast stimulation was carried out instead of using synthesis oxytocin in the Contraction Stress Test (CST), literature studying the relation between breast stimulus and plasma OT level in the 1980's is scattered. CST is an examination of the response of fetal heart rate to uterine contractions. It was mainly conducted at the end of pregnancy as fetal surveillance for high-risk pregnant women to measure the amount of placental oxygen available to the fetus during labor. Amico, et al. (1986) reported that OT levels in the blood increased due to breast stimulation for several minutes during pregnancy. Meanwhile, Ross, et al. (1986) reported that nine out of 20 uterine contractions occurred by CST due to breast stimulation, but blood OT levels did not change. It is thought that these factors are influenced by the fact that blood OT is secreted in a pulsed manner and that there are individual differences in frequency and amplitude of pulses (Fuchs, et al., 1991). Furthermore, the involvement of the oxytocin receptors in the uterine muscle layer has been noted in terms of susceptibility to OT. Oxytocin receptors increased more than 50-fold from the late pregnancy to the start of labor (Fuchs, 1982; Kimura, 1996). With many oxytocin receptors present, the uterus responsiveness to OT increases, and with low concentrations of OT, the enhancing action of uterine contractions becomes stronger (Cunningham, et al., 2014). However, it has been pointed out that sensitivity of OT is decreased by the OT receptor gene polymorphism (Terkawi, et al., 2012).

OT can be collected from saliva, urine, cerebrospinal fluid, and amniotic fluid. Measurement of OT in saliva has less invasiveness to participants than other measurement methods and has attracted attention in recent years as a simple OT measurement method. In addition, OT in the saliva is considered to have a value, which is not easily influenced by pulsing of OT in the blood. A more stable measurement value is obtained by concentrating the collected saliva and then measuring with the enzyme immunoassay according to the analysis method recommended by the analysis kit (McCullough, et al., 2013).

From the above, it is confirmed that breast stimulation aimed at spontaneous onset of labor, as its effectiveness, achieves maturity of the cervix and labor within 72 hours. In addition, there are many questions about the RCT randomization method as the basis, and the most effective intervention time is unknown. Furthermore, the influence of OTR gene polymorphism has not be considered. In order to investigate the method of breast stimulation aimed at labor induction that could be adopted in the future, as a self-care strategy of pregnancy, we investigated the influence on OT in saliva resulting from breast stimulation.

Objectives

Prior to the study to investigate the method of breast stimulation for labor onset in low-risk pregnant women, we will consider the following.

1. Analyze the relationship of the following items by breast stimulation

   Primary outcome

   ① salivary oxytocin levels

   Secondary outcomes

   ② adequate uterine contractions, ③ cervical ripening,

   ④ the onset of natural labor within 72 hours after intervention
2. Investigate whether the individual characteristics of pregnant women from the following items affect salivary oxytocin and delivery outcome.
3. Investigate the feasibility of research procedures such as breast stimulation and saliva collection.

Significance of the Study

The following are expected for the outcomes and developments obtained by this research.

1. By knowing the tendency of the effects of oxytocin and oxytocin receptor genotype in saliva, it is possible to obtain suggestions for examination of the most effective intervention time of breast stimulation for the occurrence of labor.
2. In the future, if the efficacy of breast stimulation is confirmed in a comparison group with a control group or with RCT, then this might be an effective complementary alternative therapy to prevent post term pregnancy, or to reduce medical induction.

Definition of Terms

Breast simulation: stimulating the breast including the nipple using fingers and instruments.

Spontaneous labor onset: Labor onset leading to parturition without using medicines, instruments, etc.

Natural childbirth: It leads to vaginal delivery without using medicine or instruments.

Cervical ripening (using Bishop score: BS): A score of 5 items with 0 to 3 points, 13 points are full marks. The higher the score, the more the cervical is softening leading to dilation..

Adequate uterine contraction: At least three contractions lasting for 40 seconds each occurring within 10 minutes are *adequate contractions*.

Methods

Study Design

This trial was a quasi-experimental single-arm time series design.

Participants

Participants for eligible

Women were eligible to participate if they:

(1) were between 25 and 35 years of age
(2) planned to give singleton birth by spontaneous cephalic delivery
(3) were between 38 and 40 weeks of gestation
(4) were Asian and could read and write Japanese
(5) had permission from their attending obstetrician or midwife

Women were excluded from the study if they:

(1) were taking any medications related to their gestation
(2) have medical or pregnancy complications
(3) have mental illness
(4) have a medical history of Assisted Reproductive Technology treatment,
(5) had a BMI above 25 before pregnancy
(6) planned induced labor
(7) experienced prolonged pregnancy
(8) had a previous caesarian section
(9) are breastfeeding a child

Setting and Sample size

One hospital can manage parturition and have hospitalization facilities. The number of research subjects shall be about 10 to 15 women.

Recruitment for setting

Request research cooperation with facility using ‘request document to facility’ (document 1) and ‘survey procedure document’ (document 2).

If approval of research cooperation is obtained, posted at the setting at the outpatient ward (document 10) will be ‘information disclosure’ on this research.

Recruitment for participants

Extract names of pregnant women who will be recruited from outpatient medical records.

When the targeted pregnant woman visits the regular prenatal checkup after 34 weeks of gestation, explain the purpose and content of the research in the 'request document' (document 3) and the 'leaflet' (Document 4) for the participants. Target pregnant women who gave consent.

Data Collection

It will be done from May 2015.

Procedure for Conducting the Study

The intervention period is set to three days, and the duration of breast stimulation per day is 15 minutes on each side, for a total of one hour. Pregnant women will conduct stimulation with their fingers. In order to standardize the stimulation, the vaginal pressure measuring instrument that shows the strength of the stimulus and the indication of the stimulation tempo by the electronic metronome will be used. Have clothing that is easily accessible to the nipples.

For breast stimulation, use the designated nursing cape. Do not permit accompanying persons in the room. The flow of the whole study (Figure 1) and the flow of the intervention period (Figure 2) are shown.

First day

1. After visiting, measure with Bishop Score by one research collaborator.
2. Explain the flow of the day in a room where privacy can be maintained.
3. Have the question sheet 1 (Document 7) filled out. As it is 30 minutes before saliva intake, water intake is finished.
4. Collect the oral mucosa using a swab.
5. Take a break for 20 minutes. Measure the body temperature after installing the NST monitor at the semi Fowler's position.
6. During a break, researchers or research collaborators explain the method of breast stimulation. A measure of stimulation pressure (mmHg) is shown using a vaginal pressure measuring instrument (MizCure manufactured by OWMED). Have pregnant women carry out.
7. For the method of breast stimulation, massage the nipple / areola part with the fingers from the right breast to left alternately every fifteen minutes. After lubricating with

horse oil use the thumb, index finger and middle finger to grasp the nipple and rotate clockwise and counter clockwise for 15 minutes and then switch to the other nipple and do likewise. Lubricants (horse oil) are prepared by researchers and have them used for all three days of intervention. Using an electronic metronome, stimulate about 70 times per minute. Make the sound of the metronome the lowest volume setting within the range that the woman can hear. If the woman complains about the use of a sustained metronome, check the tempo of the stimulus every 15 minutes.

8. First saliva collection (pre-stimulation value).
9. Start viewing the DVD prepared by the researcher.
10. After 15 minutes of right breast stimulation, second saliva collection is performed (15 minutes after stimulation).
11. After performing the left breast stimulation for 15 minutes, collect saliva at the third time. Immediately after collection of saliva, drink water (30 minutes after stimulation).
12. Implement right breast stimulation for 15 minutes.
13. After the left breast stimulation is performed for 15 minutes, the fourth saliva collection is performed (60 minutes after stimulation)
14. Fifteen minutes after the end of breast stimulation, the fifth saliva collection is performed (75 minutes after stimulation).
15. Thirty minutes after the end of breast stimulation, the sixth saliva collection is performed (90 minutes after stimulation).
16. The woman wears the monitor until 30 minutes after the end of breast stimulation, this confirms that the fetus is reassuring, and then terminates monitor.
17. In the case of pre-labor pain, consult with the woman and consider whether to stay inside the hospital or go home. Even with uterine contraction, if awareness is weak, woman's return home is allowed. If woman judges to be in labor and if it can not be judged, report it to your doctor or nurse in charge of research and ask instructions.
18. After collection of the sixth saliva, woman answers questionnaire 2 (Document 8) on fatigue and pain due to breast stimulation.

Second day

1. Explain the flow of the day in the room where privacy can be maintained after coming to the hospital.
2. The first day of intervention as No.5 to 18 are carried out in the same way.

Third day

1. Explain the flow of the day in the room where privacy can be maintained after coming to the hospital.
2. The first day of intervention as No. 5 to 17 are carried out in the same way.
3. After collecting the 6th saliva, fill in questionnaire 3 (reference 9).
4. Measure the Bishop Score.

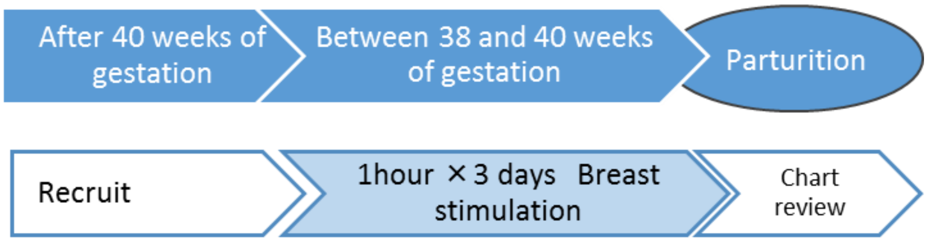

Figure 1. Flow of research

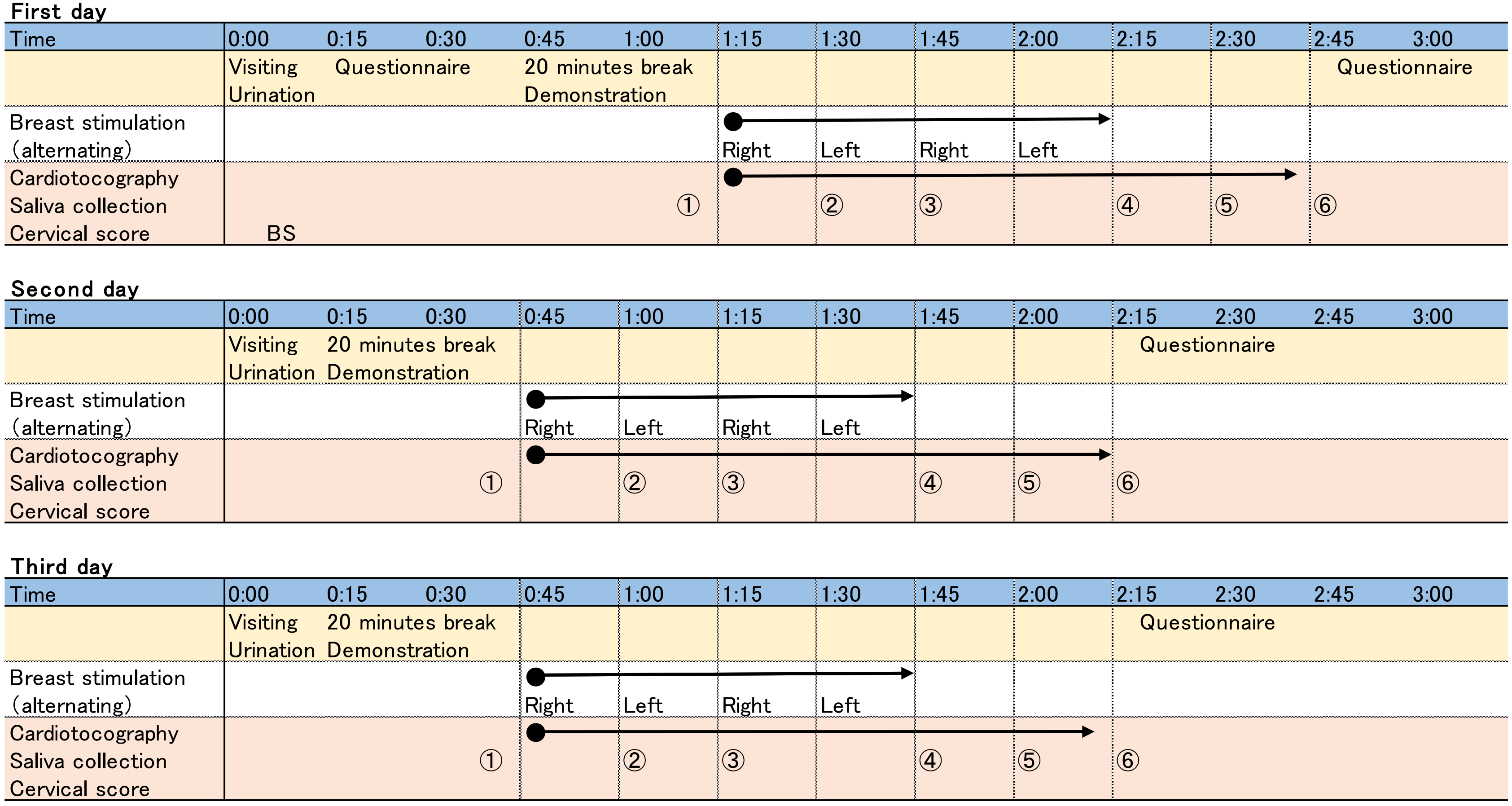

Figure 2. Flow of experiment term

Outcome Measurements

Primary outcome: Salivary oxytocin level

Collection tool and analysis

For saliva collection use 2.0 ml Eppendorf tube and straw. Oxytocin value measurement is performed by ELISA method at Professor Kazuyuki Shinohara's laboratory, Nagasaki University Graduate School.

Collection conditions

Since salivary oxytocin level may be influenced by various factors such as eating and drinking and activities, it is considered preferable to measure the condition consistently. Therefore, the following conditions are set.

① Prohibition of sexual intercourse and alcohol intake the day before research participation

② On the day of study participation, take breakfast

③ The collection time is between 10:00 and 15:00

④ From 1 hour before saliva collection, smoking, brushing, eating and drinking are prohibited (only water can be ingested)

⑤ Finish ingesting water 30 minutes before sampling

⑥ Normal temperature

⑦ No use of lipstick

Collection environment

During the research period, use a measurement-dedicated space installed in the cooperating facility for research. Space takes care to keep privacy.

Collection method

In this study, saliva collection (passive drool) by straw is performed.

① Tilt face slightly forward, close your mouth, wait for 1 to 3 minutes for saliva to spontaneously accumulate in your mouth. On the tube, describe the ID of the subject, the collection date and time, and the number of measurements.

② After 3 minutes, to salivate in the Eppendorf tube to the level of 1.0 ml. using a straw. Be careful at this time to make saliva and not foam.

③ If the amount of saliva is insufficient, wait for a few more minutes.

④ When saliva accumulates, throw away the straw and close the lid tightly until it clicks. Dispose of spent straw.

⑤ The researcher or research collaborator preserves in the cold storage containing the refrigerant
⑥ Freezing specimens in a -80 degree refrigerator
⑦ When a certain number of samples gather, send the specimen to Nagasaki University

Secondary outcome

Polymorphisms of oxytocin receptor

Collection tool and analysis

To analyze OTR gene polymorphism, oral mucosa is collected. The each specimen is collected using a cotton swab on the oral mucosa (hereinafter cotton swab), and placed in a 1.5 ml tube, that is placed inside a nylon bag with zip. Analysis is conducted under the guidance of Takuya Shuo, senior researcher at St. Luke's Research Laboratory.

Collection environment

The measurement condition shall be 1 hour or more after meal. To prevent other gene contamination, care should be taken not to touch the tip of the swab and to prevent contamination.

Collection method

(1) Describe collection date and ID in nylon bag with zip
(2) Take out one swab.
(3) Put the tip of the cotton swab in the back of the mouth, spread and with a normal force, rub the right oral mucosa of the for about 30 seconds (30 times).
(4) Using the same swab, the oral mucosa of the left cheek is taken in the same way.
(5) Insert the rubbed tip into the tube and place the whole swab into the container.
(6) With the cover of the tube open, let it air dry for more than 2 hours in a cool and dark place with good ventilation.
(7) After drying, place in a nylon bag with zip and seal it.

Questionnaire

State-Trait Anxiety Inventory; STAI

STAI is a measure created by Spielberger et al. (1970) and divides anxiety into state anxiety and trait anxiety. STAI has been validated as an instrument to measure anxiety in perinatal women (Meades et al., 2011). In pregnant women, moderate inverse correlation was observed between plasma OT and STAI (Alison et al., 2013). In this study, we set a cut-off value and used it to analyze the relationship with OT secretion in saliva

due to the strength of anxiety.

STAI, Shimizu et al. (1981) created the Japanese version and validity and reliability have been confirmed. In this research, the Japanese version STAI by Shimizu et al. (1981) is used and 20 items for state anxiety and trait anxiety are implemented on a 4-level Likert scale from 1=*not at all* to 4=*very much so*.

Practicality and acceptability of experimental methods

After the intervention, participants answer the questionnaire created by the researcher. Questionnaire 2 (Document 8) to be conducted after the end of the intervention day 1 and 2 is the same. Have them answer using Visual analog scale (VAS) regarding breast stimulation and fatigue of saliva collection. Questionnaire sheet is required to takes about 3 minutes.

After the end of the intervention of 3 days, 'question paper' 3 (Document 9) is done. There are two question sheets, which takes about 5 minutes. As with the questionnaire, VAS was used for breast stimulation and fatigue feeling of saliva collection, and a five-level Likert scale was used for the question of the strength of stimulation and tempo. In addition, we used a 4-level Likert scale: 1 (*Very much*) to 4 (*I do not think so*) about discomfort, easy-to-understand guidance, whether you would like to do it even at the next pregnancy, or want to provide information to friends. Besides that, ask questions about the environment where intervention was carried out.

Participant background

Collected contents are age, height, weight, non-pregnancy weight, non-pregnant BMI, obstetrics history, past labor situation, scheduled delivery date, number of weeks of pregnancy, whether participant's mother was post-term with participant, history of folic acid ingestion, medication history, occupation, maternity leave the number of gestational weeks that began to acquire, marital status, living with partner, educational background, household income.

During experiment data

Evaluation of cervical ripening by Bishop Score. Bishop Score is an internal examination method, and it is easy for errors to occur by evaluators, so we will evaluate with only one research collaborator (other than researcher).

Also, using a fetal heart rate monitor, observe uterine hyperstimulation and fetal distress. Confirm the subjective contraction of the uterus after the visit on the 2nd day

and 3rd day intervention.

Delivery outcome

Data on childbirth will be collected from medical records after childbirth. The contents of collection include the spontaneous onset of labor within 72 hours after intervention, the induction / augmentation of labor, the number of weeks of delivery, the mode of delivery, the delivery abnormality, the duration time for delivery, the amount of bleeding at birth, the baby weight at birth, gender, Apgar score (1 and 5 minutes), presence and degree of amniotic fluid turbidity, hospitalization to NICU.

Analysis

Calculate the basic statistics of each variable. To analyze relationships and differences between variables, we use chi-square test and t-test. For analysis, use statistical software IBM SPSS Statistics version 20.0 for Windows and make it significant at two-tailed test $p < 0.05$. Also, collecting and analyzing data through all research process receives supervision by experts in the field of maternity and midwifery, and statistical experts.

The missing value of the scale is supplemented with the average value. Other missing values are treated as defects as they are. When reliability of the inspection value can not be obtained due to abnormality of the measurement sample, it is treated as missing data.

The contents of the analysis are mainly as follows.

1. Analysis of the subject's baseline (age, obstetrics, educational background, household income, BMI, number of gestational weeks at intervention)
2. Calculation of average value, median value, range, etc. of oxytocin value in saliva for each measurement point (Changes in absolute values between individuals)
3. Comparison of data before and after intervention (Bishop Score before and after the intervention, adequate uterine contraction, uterine hyperstimulation, significant fetal bradycardia)
4. Calculation of average value, median value, range, etc. of maternal outcome (number of weeks of delivery, method of starting delivery, mode of delivery, duration time for delivery, amniotic fluid turbidity, amount of bleeding at delivery)
5. Calculation of average value, median, range, etc for outcome of infants (Apgar score, birth weight, fetal dysfunction, entry to NICU)

6. Relationship between salivary oxytocin level and each item (onset of labor within 72 hours after intervention, cervical ripening degree, adequate uterine contraction, other outcomes)
7. Relationship between oxytocin receptor genotype and each item (salivary oxytocin value, onset of labor within 72 hours after intervention, cervical ripening degree, pronounced uterine contraction, other outcomes)
8. Relationship between Japanese version STAI and salivary oxytocin value, delivery outcome

Ethical consideration

Protection of human rights in intervention studies

In accordance with the "Ethical Guidelines for Research on Medical Science for Human Beings", we will comply with this research plan and assure human rights protection.

1) How to obtain consent from the participants.

Researchers or research collaborators fully explain the contents of this research by using the consent explanation document that the ethics committee approved in advance to the subject. Please give the participants enough time to judge the opportunity to ask questions and whether to participate in this research and confirm that you have understood the contents of this research well and participate in this research before starting the research and with respect to the participants obtaining written consent by free will (Document 5). For the consent form, the researcher or research collaborator who explained and the participants who consented both sign and date, and the original is kept by the researcher, and the consent form (copy) is given to the participants.

When a change is made to the research plan that will affect the consent of the participant, we promptly provide information to the participants, confirm the intention of the participants as to whether or not to continue to participate in the research, Revise the consent explanation document etc. with the approval of it, and obtain re-agreement to the participants.

2) Profit to be provided to participants

For the participants who participated in the research, pay 9000 yen (3000 yen per day) for 3 days as a remittance by direct deposit.

3) Danger and disadvantage arising from research cooperation and

consideration for it

Possible adverse events in this study are premature rupture due to breast stimulation or excessive contraction of the uterus and its accompanying fetal dysfunction. In addition, it is possible that skin symptoms such as allergic symptoms and itching may occur due to breast stimulation using a lubricant.

Upon intervention, fetal heart beat monitoring is performed, and when adverse events are admitted during the present study, report to the doctor immediately and take appropriate measures and record.

4) Treatment and supplementation in case of accident

If an adverse event occurs due to the implementation of this research and health hazards occur to the subject, the research director and the medical institution in charge will respond so that appropriate treatment and other necessary measures can be taken. Health insurance is applied to the treatment etc. to be provided, and compensation by other money is not carried out. However, skin symptoms such as allergic symptoms and itching due to breast stimulation using lubricant are compensated by researcher's compensation insurance.

5) Ensure voluntariness for research subjects

Participation in this research is determined by the voluntary intention of the woman. Even after agreeing once, you can withdraw it at any time. Explain that there is no disadvantage due to non-participation in the research. Confirm consent to participate in the research by signing the consent form, and when giving the consent form, we also hand out the withdrawal document (document 6).

6) Presence of expenses incurred by research cooperation and a person to burden

As additional expenses for participation in this research, there is a possibility that transportation expenses will be required to visit the cooperating facilities. This transportation fee shall be included in the remuneration to the participant. In addition, the examination expenses required for this research are covered by research expenses of researchers. Therefore, participation in this research will not increase the burden of medical expenses of the subject. In addition, examination expenses, examination fee, and drug expenses to be carried out in ordinary medical treatment are carried out in the subject's insurance medical treatment.

Protection of personal information

1) All personal data related to this research are handled as anonymous by registration number and are not used for purposes other than this research. The researcher prepares a correspondence table of the medical record number, name, and date of birth and research subject identification code set at the facility. Researchers manage the data, but research collaborator manage the correspondence chart.

2) The results of this research will be published in academic journals etc. When announcing to the outside such as academic societies, papers, and conferences, participants data remains anonymous so that individuals are not specified. Also, when using a personal computer at the stage of analysis, manage the password thoroughly so that only the researcher can access. When seeking disclosure of research results, it is possible to disclose by contacting the researcher's contact information described in the research cooperation instruction.

3) Data collected in this research (data collection paper, flash-drive memory, etc.) shall be stored for five years from the end of data collection. After that, all data will be erased in a state that cannot be recovered or it will be shredded and destroyed with a shredder or the like.

4) In the next study after the completion of the preliminary study, if there is no major change in the intervention method, we will plan the secondary use of the data collection by adding the data obtained in this survey to next research. For that purpose, I also explain the point to the eligible woman and get consent. When conducting secondary research, submit the revised proposal to Research Ethics Review Committee and conduct it only when it is approved.

5) When employing a research collaborator, obtain the consent to pledge to not disclose, leak information or not use any information without permission, (Document 11) about the information that can be found in the research.
